# MoScd2 is involved in appressorium formation and pathogenicity via the Pmk1 MAPK pathway in *Magnaporthe oryzae*

**DOI:** 10.1007/s44297-023-00001-0

**Published:** 2023-08-10

**Authors:** Li-Xiao Sun, Hui Qian, Ming-Hua Wu, Fu-Cheng Lin, Xiao-Hong Liu

**Affiliations:** 1 Key Laboratory of Biology of Crop Pathogens and Insects of Zhejiang Province, Institute of Biotechnology, Zhejiang University, Hangzhou, 310058 China; 2 State Key Laboratory for Managing Biotic and Chemical Treats to the Quality and Safety of Agro-Products, Institute of Plant Protection and Microbiology, Zhejiang Academy of Agricultural Sciences, Hangzhou, 310021 China

**Keywords:** MoScd2, Spores, Appressorium, Pathogenicity, MAPK signal pathway

## Abstract

Rice blast, known as rice “cancer”, is caused by *Magnaporthe oryzae* and is particularly serious in Asian and African rice regions. China is also a frequently occurring region of rice blast. Rice blast not only seriously threatens the yield and quality of rice but also affects food security in China. In *M. oryzae*, the Mst11-Mst7-Pmk1 MAPK signaling pathway mediates pathogenicity by regulating the formation of appressorium and the development of infection hyphae. Stomatal cytokinesis defective 2 (Scd2, also called Ral3 or Bem1) is a component of the Scd complex, which has been proven to be closely related to the MAPK signaling pathway. However, its biological roles in *M. oryzae* remain elusive. Here, we identified MoScd2, a homologous protein of *Schizosaccharomyces pombe* Scd2, and preliminarily revealed its role in the development of rice blast fungus. We found that MoScd2 was involved in colony growth, sporulation, spore morphology, spore germination, appressorium formation, turgor in appressoria, mobilization of glycogen from spores to appressoria and pathogenicity. The deletion of MoScd2 resulted in a reduction in Pmk1 and Mps1 phosphorylation levels. In addition, MoScd2 was confirmed to interact with MoMst50, which is a key component of the MAPK signaling pathway in *M. oryzae*. In summary, MoScd2 was involved in the MAPK signaling pathway of *M. oryzae* via interaction with MoMst50 to participate in the influence of pathogenicity. In addition, MoScd2 also influences *M. oryzae* pathogenicity by participating in autophagy.

**Supplementary Information:**

The online version contains supplementary material available at 10.1007/s44297-023-00001-0.

## Introduction

Rice is one of the three major food crops in the world and is the diet of more than half the global population [13, 22]. Rice blast, known as the "cancer" of rice, is one of the most devastating rice diseases in the world; it can occur throughout rice production and seriously threatens the yield and quality of rice [10]. *M. oryzae* is the pathogenic fungus of the rice blast, which completes the disease cycle through conidia. Under suitable conditions, conidia germinate and form appressorium, which is a main “weapon” for rice blast [14, 38]. Subsequently, glycogen and droplets are transferred from spores to the appressorium and degraded to produce glycerol, which forms a turgor pressure of up to 8.0 MPa. Driven by a huge turgor pressure, the appressorium forms an infection peg for penetrating the leaf cuticle to produce invasive hyphae (IHs) [9, 10, 40]. After 5–7 days, fusiform lesions can be found on the surface of rice leaves, which contain a large number of conidia, and a new infection cycle occurs [17].

Scd2, also called Ral3 or Bem1, a component of the Scd complex, with two Src homology 3 (SH3) domains, a PhoX homologous (PX) domain and a PhoX and Bem1 (PB1) domain, is important for exocytosis, cytokinesis, mating and morphogenesis [6, 7, 28, 37]. The SH3 domain is usually a single copy in proteins but a double copy in adaptor proteins. Studies have suggested that the SH3 domain in adaptor proteins might be a site for proline-rich sequences to bind and use tyrosine protein kinases to transmit signals from the cell surface to downstream effector proteins [25]. The PX domain can bind to the SH3 domain or phosphoinositide [1, 32]. The PB1 domain recognizes regions containing PhoX and Cdc (PC) motifs to bind specific target proteins [15, 39]. In *Arabidopsis thaliana*, the Scd complex (Scd1 and Scd2) functions with the exocyst and RabE1 in exocytosis and cytokinesis [28]. In *Saccharomyces cerevisiae*, Bemlp links GTPase activity to Ste20p, the kinase promoter of mitogen-activated protein kinase (MAPK) cascades, through interactions with Ste5p and Cdc42p [19, 27, 31]. In *S. pombe*, the Scd complex, Cdc42 and Ras1 function as protein complexes and are required for mating and morphogenesis [6, 7, 12]. Scd1/Ral1 is a guanine nucleotide exchange factor of Cdc42 and is regulated by Ras1. Scd2/Ral3, a scaffold protein, positively affects protein binding in the Ras1-Scd pathway. RAS proteins, consisting of Ras1 and Ras2, activate a variety of downstream signaling pathways in their GTP-bound state [4, 29, 41]. In *M. oryzae*, MoRas1/2 is located upstream of the Pmk1 MAPK signaling pathway; overactive MoRas1/2 results in improper activation of the pathway and damage to appressorium formation and penetration [20, 50].

As noted above, the biological function of Scd2 has been well validated in *S. cerevisiae* and *S. pombe* but has not been identified in filamentous fungi. In this research, we characterized MoScd2 in *M. oryzae* and preliminarily studied its function in *M. oryzae*. Phenotypic assays indicated that MoScd2 plays a role in sporulation, spore morphology, spore germination, appressorium development, turgor pressure, mobilization of glycogen from spores to appressoria, and pathogenicity. Similar to *S. cerevisiae* and *S. pombe*, MoScd2 is also involved in MAPK kinase signaling pathways, and MoScd2 affects the phosphorylation levels of MoPmk1 and MoMps1 by interacting with MoMst50. In addition, MoScd2 also influences *M. oryzae* pathogenicity by participating in autophagy.

## Results

### Identification of MoScd2

**Fig. 1:**
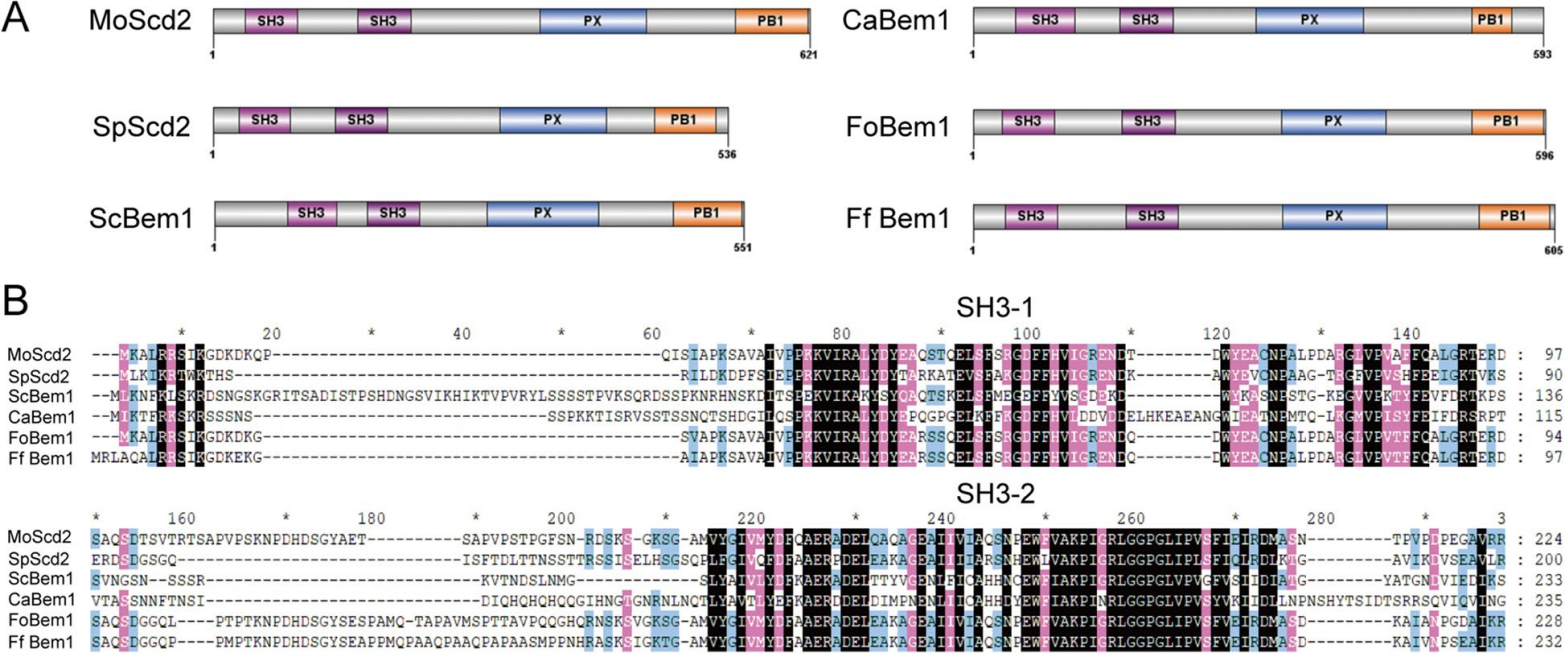
Comparison of Scd/Bem1. **A** Conserved domains of MoScd2, SpScd2, ScBem1, CaBem1, FoBem1, and FfBem1. **B** Sequence alignment of SH3 domains

MoScd2 (MGG_01702) was identified by searching against the *M. oryzae* genome database with *S. pombe* Scd2 as a quary. Using NCBI to compare homologous proteins in *M. oryzae*, *S. cerevisiae*, *S. pombe*, *Candida albicans*, *Fusarium oxysporum*, and *F. falciforme*, it was found that they are very conserved, consisting of two SH3 domains, one PX domain, and one PB1 domain (Fig. 1A). Further comparison showed that the first SH3 domain had high homology between proteins from different species, and the second SH3 domain also had high homology. However, the low homology between the first and second SH3 domains in each protein may indicate functional differences between the two SH3 domains (Fig. 1B).

### MoScd2 is involved in the growth and pathogenicity of *M. oryzae*

**Fig. 2:**
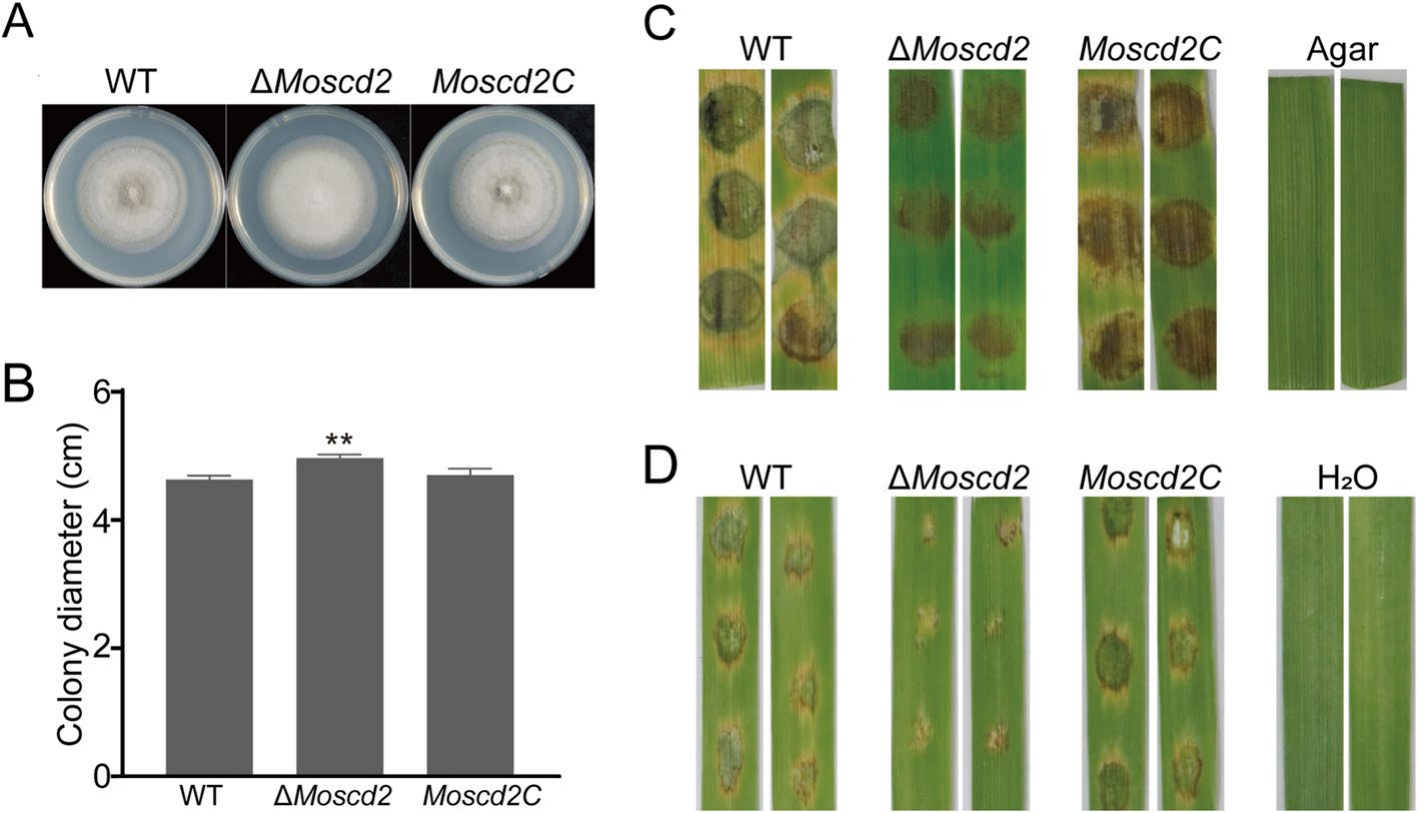
MoScd2 was involved in the growth and pathogenicity of *M. oryzae*. **A** WT, ∆*Moscd2* and *Moscd2C* were cultured on CM, alternating at 25 °C for 16 h in light and 8 h in darkness for 8 days to observe colony morphology. **B** Colony diameters of WT, ∆*Moscd2* and *Moscd2C*. Tukey’s test was carried out for significant differences: **P < 0.01, *P < 0.05. **C** Mycelial plugs from WT, ∆*Moscd2* and *Moscd2C* were placed on detached barley leaves to detect pathogenicity. **D** Spore solution with a concentration of 5 × 10^4^ conidia/ml was placed on detached barley leaves to detect pathogenicity

To characterize the functions of MoScd2, the deletion mutant Δ*Moscd2* was generated by a high-throughput target‒gene deletion strategy [26] (Fig. 2A, Fig. S1). Compared with WT and the complemented strain *Moscd2C*, Δ*Moscd2* grew slightly faster (Fig. 2B). Next, we detected the pathogenicity of WT, Δ*Moscd2* and *Moscd2C* on detached barley leaves. Mycelial plugs of WT, Δ*Moscd2* and *Moscd2C* were incubated on detached barley leaves for 4 days, and small, restricted lesions were produced by Δ*Moscd2* (Fig. 2C). Spore suspensions (5 × 10^4^ spore/ml) were sprayed onto detached barley leaves for 4 days, and small, restricted lesions were also produced by Δ*Moscd2* (Fig. 2D). Thus, the absence of MoScd2 leads to a decrease in the pathogenicity of *M. oryzae*.

### MoScd2 is important for sporulation, spore morphology and spore germination in *M. oryzae*

**Fig.3:**
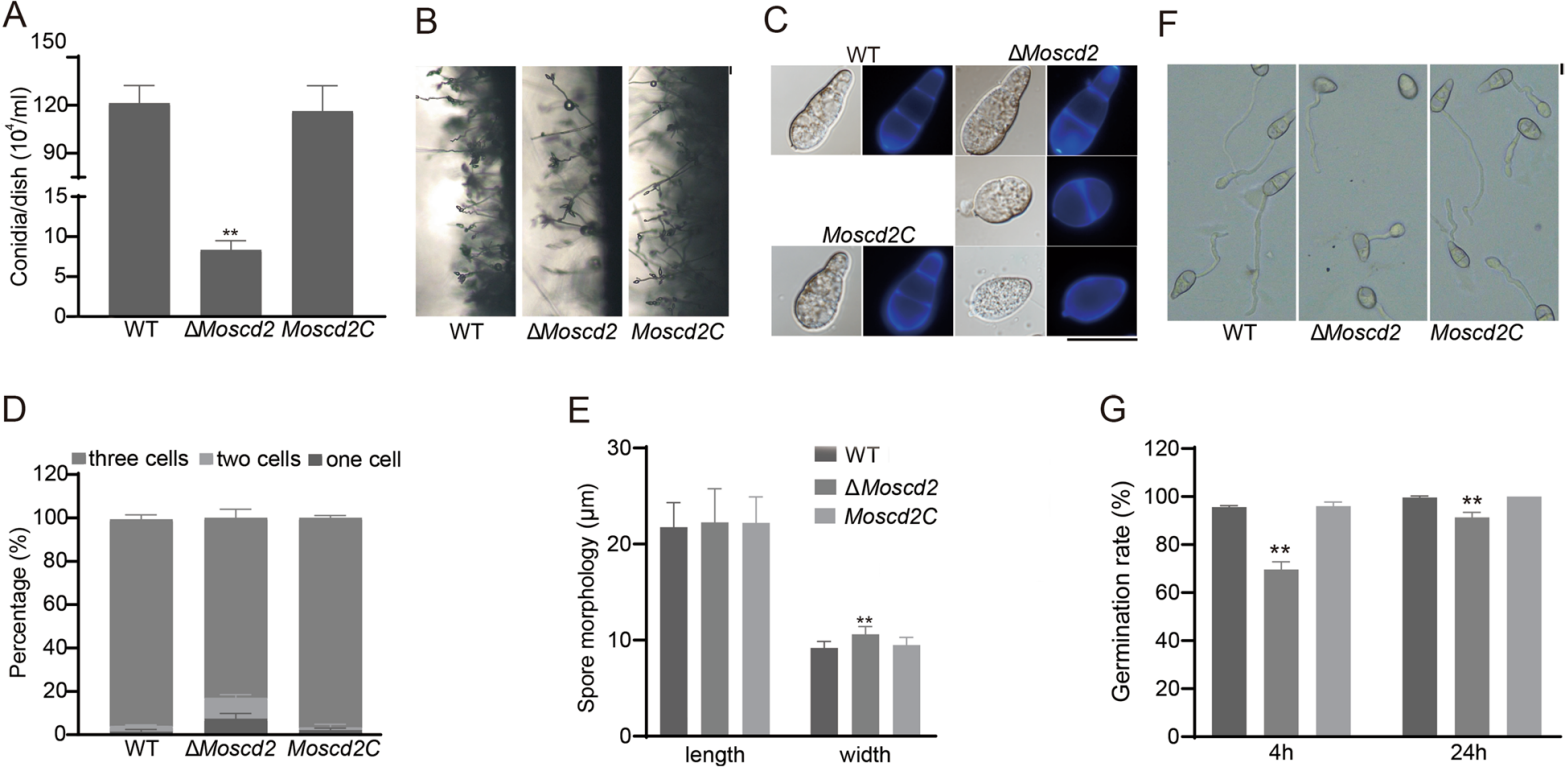
MoScd2 was important for sporulation, spore morphology and spore germination in *M. oryzae.* **A** Conidiation of WT, ∆*Moscd2* and *Moscd2C*. **B** Conidiophores of WT, ∆*Moscd2* and *Moscd2C* were observed under a light microscope. Bar = 20 μm. **C** Spore morphology of WT, ∆*Moscd2* and *Moscd2C* and conidia septum were visualized by CFW (Sigma‒Aldrich). Bar = 10 μm. **D** The three types of spores were quantified. **E** Statistical analysis of the length and width of conidia of WT, ∆*Moscd2* and *Moscd2C*. **F** Spore germination of WT, ∆*Moscd2* and *Moscd2C* at 4 hpi. Bar = 10 µm. **G** Spore germination rate of WT, ∆*Moscd2* and *Moscd2C* at 4 hpi and 24 hpi

*M. oryzae* is the pathogenic fungus of the rice blast, which completes the disease cycle through conidia. As shown in Fig. 2C and D, the absence of MoScd2 leads to a decrease in the pathogenicity of *M. oryzae*, and we are very curious whether the decrease in pathogenicity of Δ*Moscd2* is caused by abnormal spores. We took statistics on sporulation. WT was 1.21 ± 0.11 × 10^6^ spores/ml^−1^, *Moscd2C* was 1.16 ± 0.16 × 10^6^ spores/ml^−1^, and Δ*Moscd2* was 0.08 ± 0.01 × 10^6^ spores/ml^−1^, which is approximately 1/15 of WT and *Moscd2C* (Fig. 3A). Moreover, the number of conidia in a conidiophore in Δ*Moscd2* was also lower than that in WT (Fig. 3B). In the process of taking statistics on sporulation, we found that the spore morphology of Δ*Moscd2* was different from that of WT and *Moscd2C*. Interestingly, spores with one septum or no septa in Δ*Moscd2* were approximately 17%, significantly more than those in WT (approximately 4.2%) and *Moscd2C* (approximately 3.4%) (Fig. 3C, D). Comparison of the morphology of conidia produced by WT, Δ*Moscd2* and *Moscd2C* indicated that the width of Δ*Moscd2* was approximately 10.61 μm, significantly wider than that of WT and *Moscd2C* (approximately 9.18 μm and 9.50 μm, respectively) (Fig. 3E). In addition to differences in spore morphology, germination is also affected by the deletion of *MoSCD2*. As shown in Fig. 3G, we counted the rate of spore germination at 4 hpi and 24 hpi. The rate of spore germination in Δ*Moscd2* finally stabilized at approximately 91%, while that in WT and *Moscd2C* basically approached 100%.

Taken together, the phenotypic analysis showed that Δ*Moscd2* was different in colony growth, sporulation, spore morphology, and spore germination compared with WT and *Moscd2C* and played an important role in pathogenicity.

### MoScd2 affects turgor pressure in appressoria and mobilization of glycogen from spore to appressoria

**Fig. 4:**
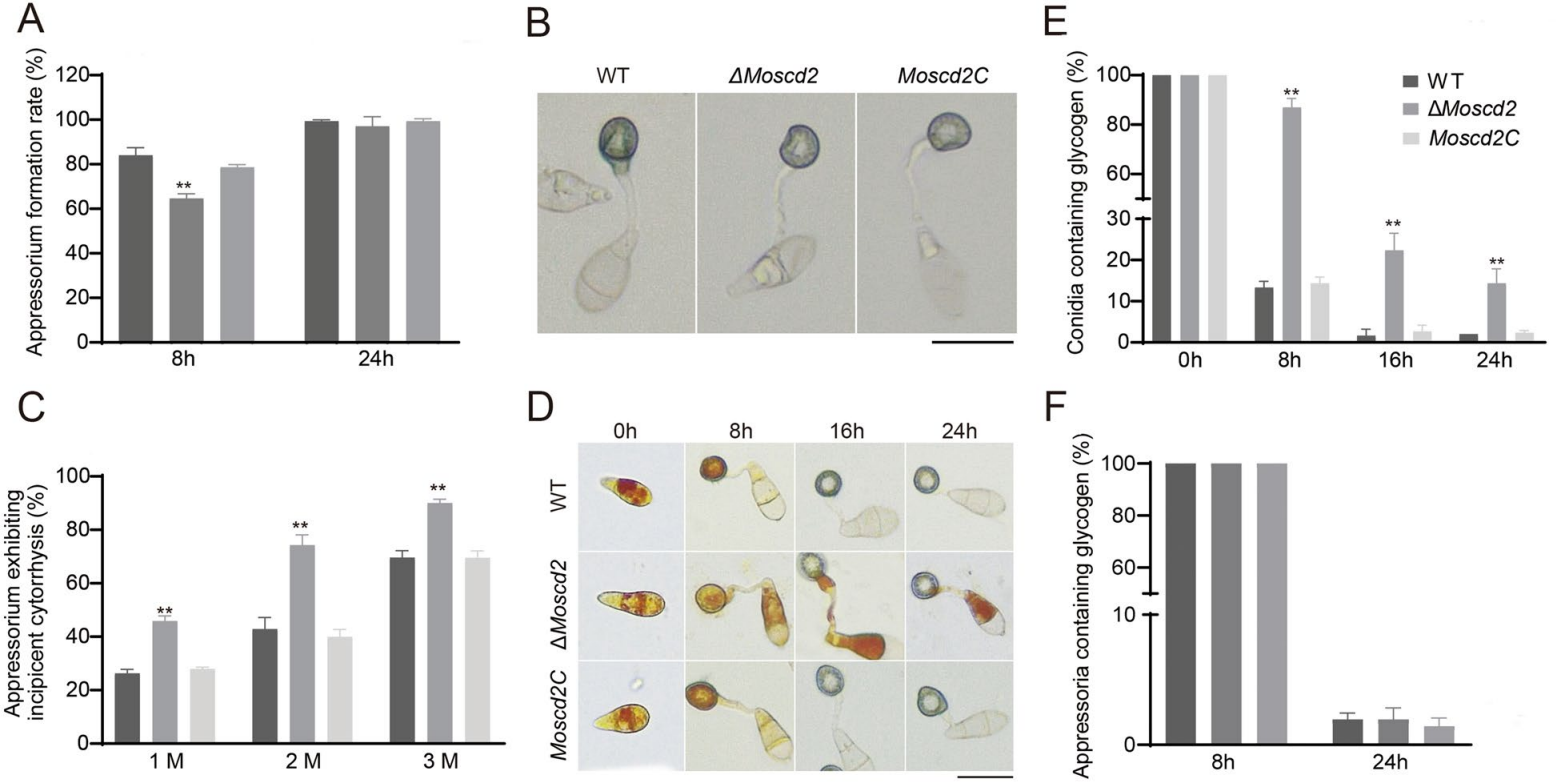
MoScd2 affected turgor pressure in appressoria and mobilization of glycogen from spores to appressoria in *M. oryzae*. **A** Appressorium formation of WT, ∆*Moscd2* and *Moscd2C*. **B** Collapse of appressoria with 1.0 M glycerol concentration. Bar = 20 μm. **C** Collapse rates of appressoria exposed to 1.0, 2.0 and 3.0 M glycerol solutions. **D** Mobilization of glycogen from spores to appressoria in WT, ∆*Moscd2* and *Moscd2C*. Bar = 20 μm. **E** Mobilization of glycogen in conidia. **F** Degradation of glycogen in appressoria

Appressorium, which produces a turgor up to 8.0 MPa, is a powerful weapon for *M. oryzae* to infect rice and is of great significance to the pathogenicity of rice blast. As shown in Fig. 4A, appressorium development was delayed in Δ*Moscd2* compared with WT and *Moscd2C*. However, when the time was extended to 24 h, this defect was remedied. Considering this, we speculated that the lower pathogenicity was associated with turgor pressure. Appressoria were exposed to 1–3 M glycerol to indicate the level of turgor, and as shown in Fig. 4C, the collapse rate of Δ*Moscd2* was significantly higher than that of WT and *Moscd2C*, suggesting that the appressorium turgor of Δ*Moscd2* is lower than that of WT.

In *M. oryzae*, glycerol produced by autophagic degradation of glycogen has been shown to be one of the sources of turgor [9, 11, 44]. To detect the turnover of glycogen, I_2_/KI solution was used to stain glycogen to observe the mobilization of glycogen from spores to appressoria at 0, 8, 16 and 24 hpi. As shown in Fig. 4D, E and F, glycogen is all present in the spore, and with the extension of time, glycogen is gradually transferred into the appressorium. At 16 hpi, the glycogen of WT and *Moscd2C* was almost completely transferred into the appressorium. However, approximately 22% of spores of Δ*Moscd2* still contained glycogen, and at 24 hpi, glycogen was still present in 14% of Δ*Moscd2* spores. Unlike the delayed glycogen transport from the spore to the appressorium, there was no difference in glycogen degradation in the appressorium between WT, Δ*Moscd2* and *Moscd2C*.

Combining the results of the turgor and glycogen assays, we speculated that the deletion of *MoSCD2* resulted in slower transport of glycogen from conidia to appressorium, resulting in reduced turgor.

### MoScd2 regulates the phosphorylation levels of MoPmk1 and MoMps1 by interacting with MoMst50

Studies have shown that the Pmk1 MAP kinase pathway plays a very important role in the development of appressorium and infected hyphae of *M. oryzae* [21].

**Fig. 5:**
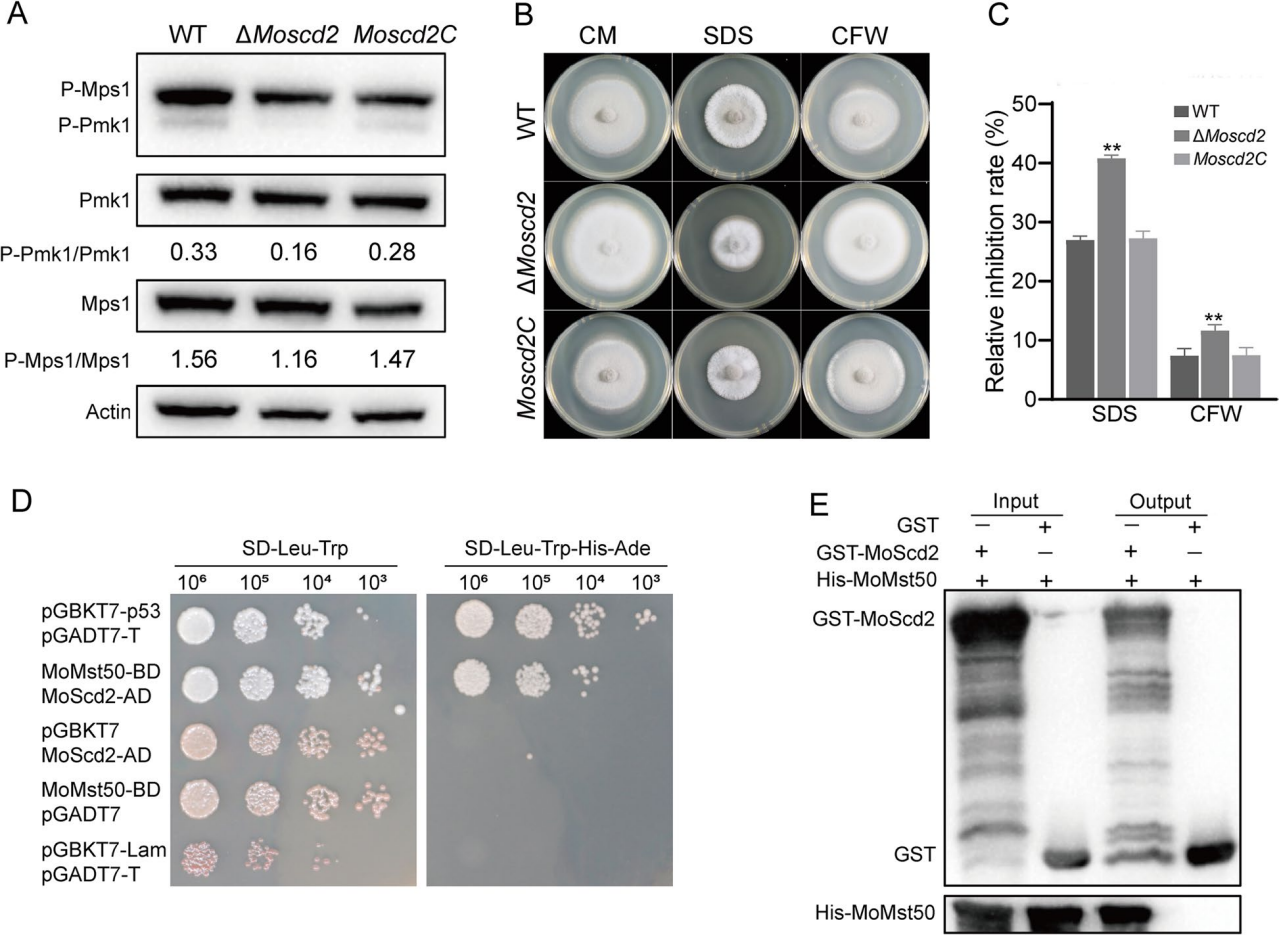
MoScd2 regulated the phosphorylation levels of MoPmk1 and MoMps1 by interacting with MoMst50. **A** Phosphorylation of Pmk1 and Mps1 in WT, ∆*Moscd2* and *Moscd2C*. **B** SDS and CFW were added separately to CM to culture WT, ∆*Moscd2* and *Moscd2C*. **C** Relative inhibition rates of WT, ∆*Moscd2* and *Moscd2C* on CM with SDS or CFW. **D** The interaction between MoScd2 and MoMst50 was verified by a yeast two-hybrid test. **E** Pull-down was used to verify the interaction between MoScd2 and MoMst50

Phenotypic assays showed that the development of the appressorium slowed, and the pathogenicity decreased in Δ*Moscd2*. Therefore, we detected the phosphorylation level of MoPmk1 in WT and Δ*Moscd2*. As shown in Fig. 5A, the phosphorylation level of MoPmk1 in Δ*Moscd2* was approximately 1/2 that of the WT and *Moscd2C*. In addition, we found that the phosphorylation level of MoMps1 in Δ*Moscd2* was slightly lower than that in WT and *Moscd2C*. MoMps1 is responsible for regulating the integrity of the *M. oryzae* cell wall, and the cell wall defects of Δ*Momps1* were obvious [8, 18, 46]. The cell wall stress test showed that Δ*Moscd2* was sensitive to the cell wall stress factors SDS and CFW (Fig. 5B and C).

Studies have shown that the connector protein Mst50 can combine with Mst11 and Mst7 to cause downstream Pmk1 phosphorylation and participate in the Mst11-Mst7-Pmk1 MAPK pathway [45]. In addition, Mst50 can also combine with Mck1 and Mkk2 to cause downstream Mps1 to be phosphorylated and participate in the Mck1-Mkk2-Mps1 MAPK pathway [21]. With further research, we found that MoMst50 is a potential interacting protein of MoScd2. The cotransfer of MoMst50-BD and MoScd2-AD into yeast cells through a yeast two-hybrid assay showed that they could not only grow normally on the SD-Leu-Trp plate but also grow normally on the SD-Leu-Trp-His-Ade plate, indicating an interaction between MoMst50 and MoScd2 (Fig. 5D). In addition to the yeast two-hybrid assay, we also verified the interaction between MoMst50 and MoScd2 through a pull-down assay (Fig. 5E).

Combining the above results, we hypothesized that MoScd2 regulated the phosphorylation levels of MoPmk1 and MoMps1 through interaction with MoMst50.

### MoScd2 was involved in autophagy

The term autophagy generally refers to the lysosomal-mediated degradation of intracellular substances from soluble proteins to intact organelles, a class of subcellular degradation pathways that play a key role in the maintenance of health in eukaryotes [30, 36]. Recently, a new viewpoint indicated that MoMkk1 could be phosphorylated by MoAtg1, thereby activating the CWI pathway involved in pathogenicity regulation [47]. As shown in Fig. 5, MoScd2 participated in the regulation of the CWI pathway through interaction with MoMst50, and we wondered whether there was a link between MoScd2 and autophagy.

**Fig. 6:**
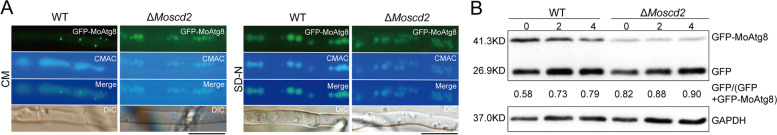
MoScd2 was involved in autophagy. **A** Localization of GFP-MoAtg8 in WT and ∆*Moscd2*. Bar = 10 µm. **B** Degradation of GFP-MoAtg8 was detected in WT and ∆*Moscd2*

At present, the most classic method to detect autophagy is the detection of Atg8 degradation. Atg8 is located on the autophagosome membrane and enters the vacuole together with the autophagosome for degradation. Therefore, by comparing the number of degraded Atg8, we can determine the number of autophagosomes formed and thus the level of autophagy. Based on the above principles, the GFP-MoAtg8 fusion protein was transferred into WT and Δ*Moscd2*. We first observed the location of GFP-MoAtg8. As shown in Fig. 6A, under CM conditions, GFP-MoAtg8 in WT was positioned around the vacuole, and GFP-MoAtg8 entered the vacuole in Δ*Moscd2*. After induction of nitrogen deficiency, GFP-MoAtg8 in WT entered the vacuole, and GFP-MoAtg8 was still localized in the vacuole in Δ*Moscd2*. To further determine the changes in autophagy in Δ*Moscd2*, we used Western blotting to detect the degradation of GFP. As shown in Fig. 6B, the autophagy level of Δ*Moscd2* was always higher than that of the WT under both CM and nitrogen deficiency conditions, which was also consistent with the results of Fig. 6A.

Combining the results of Fig. 6A and B, we can conclude that the autophagy level increased after the deletion of MoScd2.

## Discussion

At present, most of the research on Scd2 exists in *S. cerevisiae* and *S. pombe*, and research on pathogenic fungi is relatively rare. Here, we identified MoScd2, a homologous protein of *S. pombe* Scd2, in *M. oryzae*. We successfully knocked out MoScd2 in *M. oryzae* and analyzed the phenotype of Δ*Moscd2*. The phenotypic assay showed that colony growth, sporulation, spore morphology, spore germination, appressorium formation, turgor pressure in appressoria and mobilization of glycogen from spores to appressoria of Δ*Moscd2* were all affected to different degrees, which resulted in a reduction in pathogenicity.

In both *S. cerevisiae* and *S. pombe*, Scd2 belongs to the Scd complex and is related to the MAPK signaling pathway [6, 7, 12, 19, 27, 31]. Mst11-Mst7-Pmk1 MAPK mediates the pathogenicity of *M. oryzae* by regulating the formation of appressorium and the development of infected hyphae [35]. Δ*Mopmk1* failed to form appressoria, and no tissue damage was found in rice leaves infected with it [5, 40, 45]. The *MoMST7* and *MoMST11* gene deletion mutants upstream of *MoPMK1* showed similar defects in appressorium formation and the development of infected mycelia as Δ*Mopmk1* [49]. Phenotypic assays showed that the development of appressorium in Δ*Moscd2* slowed down and the pathogenicity decreased, accompanied by decreased phosphorylation levels of Pmk1, which showed great similarity with Δ*Mopmk1*, Δ*Momst7*, and Δ*Momst11*. In addition, the adapter protein Mst50 upstream of Mst11-Mst7-Pmk1 MAPK was shown to phosphorylate downstream Pmk1 after binding to Mst11 and Mst7 [45]. Phosphorylation of Pmk1 was eliminated in Δ*Momst50,* and Δ*Momst50* also showed the same defects as Δ*Mopmk1*, resulting in loss of pathogenicity in *M. oryzae* [21]. Interestingly, we identified that MoScd2 could interact with MoMst50. Combined with phenotypic assays, MoScd2 affected appressorium development by influencing the phosphorylation level of Pmk1 through interaction with MoMst50, thus affecting the pathogenicity of *M. oryzae*. Scd2 is also called Ral3 or Bem1. Although the function of Scd2/Ral3 of *M. oryzae* has not been studied, the function of PoRal2 has been described [34]. Similar to Δ*Moscd2*, Δ*Poral2* showed defects in sporulation, spore morphology, spore germination, appressorium formation, turgor pressure in appressoria and pathogenicity. The Pmk1 phosphorylation level was also reduced in Δ*Poral2*, and PoRal2 could interact with Scd1, Smo1, and Mst50, suggesting that PoRal2 also participated in the Pmk1 MAPK signaling pathway by interacting with Mst50.

In *M. oryzae*, the upstream connector protein MoMst50 can cause downstream Pmk1 to be phosphorylated to participate in Mst11-Mst7-Pmk1 MAPK after combining with MoMst11 and MoMst7 [45]; it also binds to MoMck1 and MoMkk2 to cause the phosphorylation of Mps1 downstream to participate in Mck1-Mkk2-Mps1 MAPK. Δ*Momst50*, Δ*Momck1*, Δ*Momkk2*, and Δ*Momps1* exhibited cell wall defects, and the phosphorylation of Mps1 in Δ*Momst50*, Δ*Momck1*, Δ*Momkk2*, and Δ*Momps1* also exhibited reduction to different degrees [16, 48]. In our study, Δ*Moscd2* was sensitive to cell wall stress and accompanied by decreased phosphorylation levels of Mps1. These results suggested that MoScd2 also affected Mps1 phosphorylation through its interaction with MoMst50.

Studies on *M. oryzae* showed that autophagy was closely related to its pathogenicity [23, 24]. Recently, a new viewpoint indicated that MoMkk1 could be phosphorylated by MoAtg1, thereby activating the CWI pathway involved in pathogenicity regulation [47]. Studies on Scd1 also showed a relation to autophagy, as SCD1 was described as an autophagy inducer [2]. Here, we found that the autophagic flux in Δ*Moscd2* was faster than that in WT. In addition, Δ*Moscd2* also showed defects in sporulation, spore germination, appressorium formation, turgor pressure in appressoria and mobilization of glycogen from spore to appressoria, which is similar to ATG-deficient mutants [23, 42]. Our preliminary results indicated that MoScd2 is involved in autophagy, and the specific relationship between MoScd2 and autophagy needs further study.

In conclusion, we revealed that MoScd2 in *M. oryzae* was involved in colony growth, sporulation, spore morphology, spore germination, appressorium formation, turgor pressure in appressoria and mobilization of glycogen from spores to appressoria. Furthermore, MoScd2 was revealed to be involved in regulating the MAPK signaling pathway in *M. oryzae* by interacting with MoMst50 to participate in the influence of pathogenicity.

## Materials and methods

### Strains and culture conditions

Guy11 was used as the wild-type (WT) strain, and all strains were cultured on complete medium (CM), alternating at 25 °C for 16 h in light and 8 h in darkness [3]. In the cell wall stress assay, strains were cultured on CM with 0.004% sodium dodecyl sulfate (SDS) or 30 μg/ml calcofluor white (CFW).

### Targeted gene deletion and complementation

Δ*Moscd2* was generated by a high-throughput target‒gene deletion strategy [26]. The knockout vector was pKO1B digested by *Hind*III/*Xbal*I, and the complementary vector was pKD3, with the bacterial bialophos resistance gene (BAR) digested by *Sma*I/*BamH*I. Linearized vector pKO1B, approximately 1.5-kb upstream/downstream homologous arms of the gene, cloned from *M. oryzae*, and approximately 1.3-kb hygromycin resistance gene (HPH), amplified from pCB1003, were fused by ClonExpress II One Step Cloning Kit (Vazyme, China). The linearized vector pKD3 and *MoSCD2* cloned from *M. oryzae* were fused by the ClonExpress Multis One Step Cloning Kit (Vazyme, China). The vector of successful connection was transformed into WT or ∆*Moscd2* by *Agrobacterium tumefaciens*-mediated transformation (ATMT) to obtain ∆*Moscd2* or *Moscd2C* [43]. Fluorescence observation and PCR verification were used to select the transformants that were successfully transformed. Successful replacement transformants also require verification of the number of copies by quantitative real-time PCR (qPCR) to ensure that it is a single copy. All primers used are listed in Supporting Information Table S1.

### Phenotypic characterization

WT, Δ*Moscd2*, and *Moscd2C* were cultured on CM plates for 8 days, alternating at 25 °C for 16 h in light and 8 h in darkness, to measure the colony diameter. After the measurement of colony diameter, plates with WT, Δ*Moscd2*, and *Moscd2C* were added to 3 ml of distilled water (ddH_2_O) to obtain spores, which were counted under a microscope to evaluate sporulation and observe spore morphology. Spore acquisition methods for spore germination, appressorium formation, turgor pressure in appressoria and mobilization of glycogen from spore to appressoria assays were described as above. After the spores were washed with ddH_2_O to remove impurities and nutrients, they were diluted to a concentration of 5 × 10^4^ conidia/ml. Twenty microliters of spore solution was placed on the hydrophobic surface and incubated at 22 °C in complete darkness. Spore germination was measured at 4 and 24 h, respectively; appressorium formation was measured at 8 and 24 h, respectively; turgor pressure in appressoria was measured at 24 h with glycerol solution 1.0 M, 2.0 M and 3.0 M, respectively; mobilization of glycogen from spores to appressoria was measured with I2/KI solution at 0, 8, 16 and 24 h, respectively.

For pathogenicity, mycelial plugs of WT, Δ*Moscd2*, and *Moscd2C* were added to detached 8-day-old barley leaves, alternating at 25 °C for 16 h in light and 8 h in darkness for 4 days. Spore solution with a concentration of 5 × 10^4^ conidia/ml was added to detached 8-day-old barley leaves, alternating at 25 °C for 16 h in light and 8 h in darkness for 4 days.

### Yeast two-hybrid assay and pull-down assay

The yeast strain used was Y2HGold, the positive control strains were pGADT7-T and pGBKT7-53, and the strains used to construct the vector were pGADT7 and pGBKT7. The interaction protein to be verified was cotransferred into yeast-receptive cells and cultured on SD-Leu-Trp and SD-Leu-Trp-Ade-His plates at 30°C in complete darkness for 4–6 d.

For the pull-down assay, the strains used to construct the vector were pET21 and pGEX-4 T; *Escherichia coli* was BL21 (DE3). The specific test operations are described by Qian et al. [33].

### Western blot assay

GFP-MoAtg8 was transformed into WT and Δ*Moscd2* by ATMT. WT and Δ*Moscd2* were cultured on CM plates for 8 days, alternating at 25 °C for 16 h in light and 8 h in darkness. An appropriate amount of mycelia was taken to break and placed in 150 ml liquid CM medium for shock culture for 40–48 h. For the location of GFP-MoAtg8, mycelia were stained with CMAC and then observed under a fluorescence microscope. For autophagic flux, mycelia cultured for 40–48 h were transferred to SD-N liquid medium for continuous shock culture for 2–4 h. The above proteins were extracted for detection with anti-GFP (Abcam) and anti-GAPDH (HUABIO, China).

For the phosphorylation assay, anti-phospho-p44/42 (Cell Signal Technology), anti-p44/42 (Cell Signal Technology), anti-ERK1/2 (Santa Cruz Biotechnology) and anti- Actin (ABclonal, China) were used.

## Supplementary Information

**Additional file 1.**

**Additional file 2: Table S1. **Primers used in this study.

## Data Availability

The datasets used or analyzed during the current study are available from the corresponding author on reasonable request.
