# Supplementary figures and images for: MoScd2 is involved in appressorium formation and pathogenicity via the Pmk1 MAPK pathway in Magnaporthe oryzae
Source: Crop Health. 2023 Aug 10;1(1):4. doi: 10.1007/s44297-023-00001-0 (PMC12825975; doi:10.1007/s44297-023-00001-0)

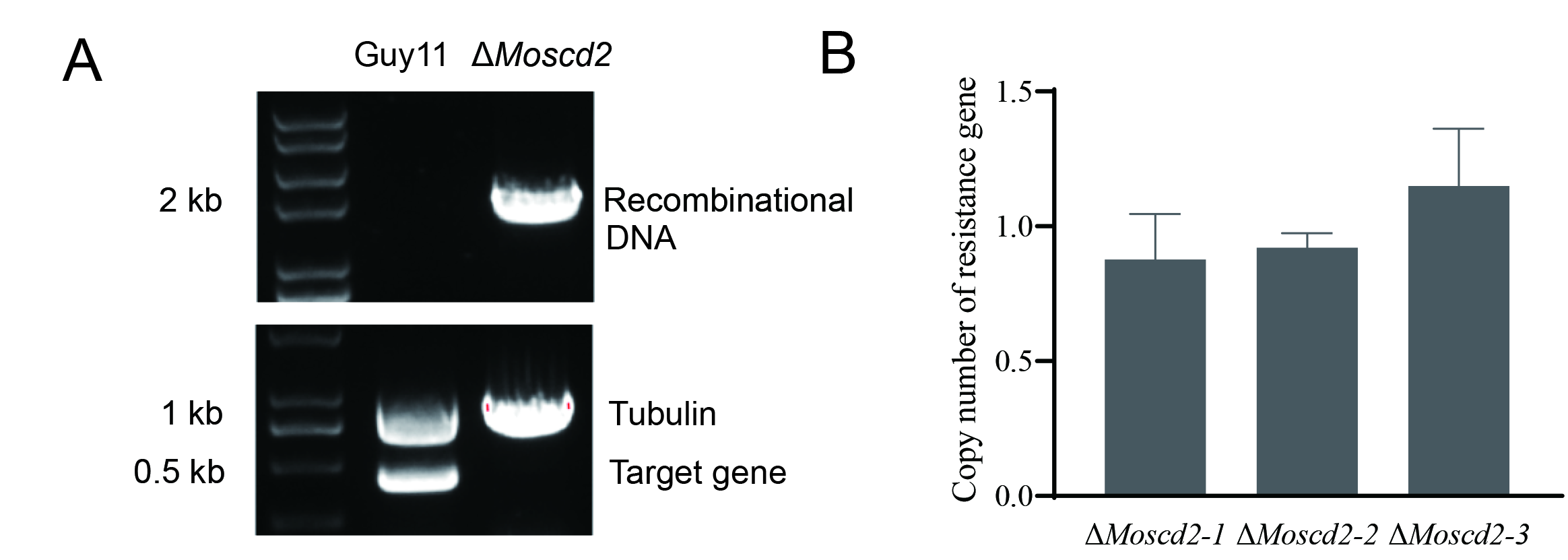

Supplement: Supplementary file 1 — Additional file 1. [file 44297_2023_1_MOESM1_ESM.tif]
