# Supplementary material for: MoScd2 is involved in appressorium formation and pathogenicity via the Pmk1 MAPK pathway in Magnaporthe oryzae
Source: Crop Health. 2023 Aug 10;1(1):4. doi: 10.1007/s44297-023-00001-0 (PMC12825975; doi:10.1007/s44297-023-00001-0)
Supplement: Supplementary file 2 — Additional file 2: Table S1. Primers used in this study. [file 44297_2023_1_MOESM2_ESM.docx]

**Table S1** Primers used in this study

| **Primer name** | **Primer** |
| --- | --- |
| **Primers for gene knock out** | |
| *MoSCD2*-UpF | GGGATCCTCTAGAGTCGAC CTGTGAGATTGAGGCTGAA |
| *MoSCD2*-UpR | TTCATTGTTGACCTCCACTA GAGACTGAATGTGGATGCT |
| *MoSCD2*-DownF | GCAAAGGAATAGAGTAGATG CGTCACAGCACAGGTATT |
| *MoSCD2*-DownR | ACGGCCAGTGCCAAGCTT GAATCCGTCGTTGAGGTT |
| *MoSCD2*-LongF | TGCCTAACTAATGACCTGTT |
| *MoSCD2*-SF | TTGCCGAGGAGAAGTATTG |
| *MoSCD2*-SR | GCCAGGTTGTTGTTGTTG |
| *MoSCD2C-F* | CAATCACAATGGCC GGATCCATGAAGGTACGTCGGTAGCTG |
| *MoSCD2C-R* | CCCTTGCTCACCATCCCGGG TATGTGATCAACGTAGAACA |
| *YHPH*-R | GATAATAATGTCCTCGTTCC |
| *HPH*-F | TAGTGGAGGTCAACAATGAATG |
| *HPH*-R | CATCTACTCTATTCCTTTGCC |
| **Primers used for qPCR** | |
| RT-*HPH*-F | ATGTCCTGCGGGTAAATAGC |
| RT-*HPH*-R | GATGCAATAGGTCAGGCTCTC |
| RT-*TUBLIN*-F | ACAACTTCGTCTTCGGTCAG |
| RT-*TUBLIN*-R | GTGATCTGGAAACCCTGGAG |
| **Primers for yeast two hybrid** | |
| *MoMST50*-BDF | CATGGAGGCCGAATTCATGAGCTTCAACACGGGGAC |
| *MoMST50*-BDR | GCAGGTCGACGGATCCTATTATTCCTCCTGGGGGATC |
| *MoSCD2*-ADF | GGAGGCCAGTGAATTCATGAAGGCCTTACGGCGATCC |
| *MoSCD2*-ADR | CGAGCTCGATGGATCCTATGTGATCAACGTAGAAC |
| **Primers for Pulldown** | |
| His-*MoMST50*-F | CAAGGTCGACAAGCTTATGAGCTTCAACACGGGGACGG |
| His-*MoMST50*-R | GTGCGGCCGCAAGCTTTATTATTCCTCCTGGGGGAT |
| GST-*MoSCD2*-F | CGCGTGGATCCCCGGAATTC ATGAAGGCCTTACGGCGATC |
| GST-*MoSCD2*-R | CTCGAGTCGACCCGGGAATTC TATGTGATCAACGTAGAAC |
